# Respiratory viral infection promotes the awakening and outgrowth of dormant metastatic breast cancer cells in lungs

**DOI:** 10.21203/rs.3.rs-4210090/v1

**Published:** 2024-04-05

**Authors:** Shi B. Chia, Bryan J. Johnson, Junxiao Hu, Roel Vermeulen, Marc Chadeau-Hyam, Fernando Guntoro, Hugh Montgomery, Meher Preethi Boorgula, Varsha Sreekanth, Andrew Goodspeed, Bennett Davenport, Felipe V. Pereira, Vadym Zaberezhnyy, Wolfgang E. Schleicher, Dexiang Gao, Andreia N. Cadar, Michael Papanicolaou, Afshin Beheshti, Stephen B. Baylin, James Costello, Jenna M. Bartley, Thomas E. Morrison, Julio A. Aguirre-Ghiso, Mercedes Rincon, James DeGregori

**Affiliations:** University of Colorado Anschutz Medical Campus; University of Colorado Anschutz Medical Campus; University of Colorado Anschutz Medical Campus; Utrecht University; Department of Epidemiology and Biostatistics, School of Public Health, Imperial College London, UK; Imperial College London; University College London; University of Colorado; University of Colorado Anschutz Medical Campus; University of Colorado Anschutz Medical Campus; University of Colorado Anschutz Medical Campus; University of Colorado Anschutz Medical Campus; University of Colorado Anschutz Medical Campus; University of Colorado Anschutz Medical Campus; Biostatistics and Bioinformatics Core, University of Colorado Cancer Center; University of Connecticut; Albert Einstein College of Medicine; Broad Institute; Johns Hopkins Medical Institutions; University of Colorado Anschutz Medical Campus; University of Connecticut; University of Colorado; Albert Einstein College of Medicine; University of Colorado Anschutz Medical Campus; University of Colorado Anschutz Medical Campus

## Abstract

Breast cancer is the second most common cancer globally. Most deaths from breast cancer are due to metastatic disease which often follows long periods of clinical dormancy^1^. Understanding the mechanisms that disrupt the quiescence of dormant disseminated cancer cells (DCC) is crucial for addressing metastatic progression. Infection with respiratory viruses (e.g. influenza or SARS-CoV-2) is common and triggers an inflammatory response locally and systemically^2,3^. Here we show that influenza virus infection leads to loss of the pro-dormancy mesenchymal phenotype in breast DCC in the lung, causing DCC proliferation within days of infection, and a greater than 100-fold expansion of carcinoma cells into metastatic lesions within two weeks. Such DCC phenotypic change and expansion is interleukin-6 (IL-6)-dependent. We further show that CD4 T cells are required for the maintenance of pulmonary metastatic burden post-influenza virus infection, in part through attenuation of CD8 cell responses in the lungs. Single-cell RNA-seq analyses reveal DCC-dependent impairment of T-cell activation in the lungs of infected mice. SARS-CoV-2 infected mice also showed increased breast DCC expansion in lungs post-infection. Expanding our findings to human observational data, we observed that cancer survivors contracting a SARS-CoV-2 infection have substantially increased risks of lung metastatic progression and cancer-related death compared to cancer survivors who did not. These discoveries underscore the significant impact of respiratory viral infections on the resurgence of metastatic cancer, offering novel insights into the interconnection between infectious diseases and cancer metastasis.

## Introduction

Breast cancer is the most diagnosed cancer in women and is the second leading cause of cancer-associated deaths in the U.S.^1^ After the initial remission, disseminated cancer cells (DCC) can stay dormant for years to decades^4^ before metastatic relapse, most commonly in lung, bone, and liver^4^. Both cell-intrinsic factors and the tumor microenvironment dictate if metastatic cells stay dormant or proliferate and form secondary tumors^5^. Importantly, perturbations of the tumor microenvironment such as with enhanced inflammation can be sufficient to increase metastasis^5^.

Viral respiratory infections are common: seasonal influenza affects over 1 billion people each year^6^ and, by March 2024, SARS-CoV-2 infection has already caused nearly 750 million cases of COVID-19^7^. Viral respiratory infections are typically associated with pulmonary inflammation, with a concomitant increase in pulmonary inflammatory cytokines such as IL-6 and interferons (IFN), and an expansion of immune cells, including neutrophils, macrophages, and T lymphocytes^2,3^. Such inflammatory mechanisms, specifically involving IL-6/signal transducer and activator of transcription 3 (IL-6/STAT3)-signaling^8,9^, neutrophils and neutrophil extracellular traps^10^, and the CD4^+^ cell/macrophage axis^11^, have been identified as regulators of metastatic processes in cancer.

The observation that death rates from cancer rose in the first two years of the COVID-19 pandemic^12^, not fully accounted for by COVID-19 deaths or delayed screening and treatment, prompts a critical hypothesis - that pulmonary viral infections, including SARS-CoV-2, increase cancer deaths by triggering the development of metastases from dormant cancer cells. We sought to test this hypothesis through a dual approach: examining the effects of viral respiratory infections (influenza virus and SARS-CoV-2) on breast cancer dormancy in mouse models and correlating SARS-CoV-2 infection among cancer survivors to metastatic progression and cancer mortality.

## Materials and Methods

### Mouse strains, influenza virus infection, and antibody treatments

Transgenic mouse models of breast cancer, using mammary tumor virus (MMTV) long terminal repeats, are widely used. In brief, MMTV-PyMT and MMTV-erbB2/neu/HER2 (MMTV-Her2) mice express the oncogenes Polyoma virus Middle T antigen (PyMT) and rat *Erbb2* (encoding Her2), respectively, upstream of the MMTV promoter which confers expression in the mammary epithelium, as described elsewhere^10,13,14^. The MMTV-PyMT transgene is congenic in the FvB background, and the MMTV-Her2 transgene is congenic in the FvB and C57Bl/6 backgrounds. MMTV-Her2 mice (FvB) were crossed with IL-6 KO mice as described^9,15^.

Eight-week-old MMTV-PyMT and 12- to 14-week-old MMTV-Her2 mice were infected with 500 EIU Puerto Rico A/PR/8/34 H1N1 influenza A (IAV) through intranasal administration in 50 ml PBS. For viral administration, mice are anesthetized using 5% induction isoflurane and 2% maintenance, performed with a SomnoFlo Low-Flow electronic vaporizer machine in an induction chamber. After ensuring adequate anesthesia with slow and deep breathing, droplets of viral fluid are placed on the mouse’s nostrils. The mouse inhales the fluid through the nostrils. Once the fluid has been inhaled, the mouse is placed on a heating pad to recover.

For immune cell depletion experiments, mice were injected intraperitoneally with rat IgG as control (MP Biochemicals; cat# MPBio 0855951; Singapore), 100 mg anti-CD4 (Bio X cell, clone GK1.5; cat# BP003–1; Lebanon, NH) (Sup. Fig. 7), or 100 mg anti-CD8 (Bio X cell, clone2.43; cat#; Lebanon, NH) 1 day before IAV infection and every 6 days afterward, or 200 mg anti-Ly6G (Bio X cell, clone 1A8; cat#BP0075–1; Lebanon, NH) on the day of the flu infection, then 24 hours and every other day afterwards, until being euthanized. The University of Colorado Institutional Animal Care and Use Committee (IACUC) reviewed and approved all animal experiments, which were conducted in accordance with the NIH Guidelines for the Care and Use of Laboratory Animals.

### SARS-CoV-2 MA10 propagation

Mouse-adapted SARS-CoV-2 MA10 (BEI Resources NR-55329) was propagated in Vero E6 cells (ATCC CRL-1586) as previously described^16^. Briefly, low passage Vero E6 monolayers were inoculated at an MOI of 0.01 with SARS-CoV-2 MA10. When Vero E6 monolayers exhibited 70–75% CPE (2–3 days post inoculation), supernatants were collected, clarified by centrifugation, supplemented with an additional 10% FBS, aliquoted and stored at −80°C. SARS-CoV-2 titers were determined by plaque assay on Vero-E6 cells. Vero-E6 cells were maintained at 37°C in Dulbecco’s Modified Eagle medium (DMEM, HyClone 11965–084) supplemented with 10% fetal bovine serum (FBS), 10 mM HEPES (pH 7.3) and 100 U/mL of penicillin-streptomycin.

### SARS-CoV-2 MA10 infection of mice

C57BL/6J MMTV-Her2 mice at 14–19 weeks of age were anesthetized by intraperitoneal injection (i.p.) with a mixture of ketamine (80 mg/kg) and xylazine (7.5 mg/kg) in a volume of 100–200 uL. Fully anesthetized mice were inoculated intranasally (i.n.) with 10^4^ PFU of SARS-CoV-2 MA10 diluted in PBS supplemented with 1% bovine calf serum by administration of 25 uL of inoculum in each nostril for a total volume of 50 uL. Mouse weights were collected daily for 15 days, and mice inoculated with SARS-CoV-2 MA10 exhibited weight loss beginning at 2 dpi, with greatest loss achieved at 3–4 dpi as previously reported^16^. As controls, C57BL/6J MMTV-Her2 mice were mock inoculated with 50 uL of PBS/1% bovine calf serum.

### Immunohistochemistry (IHC) and immunofluorescence (IF) staining

Lungs and mammary glands were collected and fixed in 10% neutral buffered formalin overnight, transferred to 70% ethanol the next day, and then embedded in paraffin. Tissue was sectioned (5 μm) and used for IHC and IF. Slides were deparaffinized in three incubations of 15 min in Histo-clear (Fisher Scientific, cat# 50–899-90147; Hampton, NH) then descending 10-min ethanol incubations: three at 100%, followed by 95%, and 70% followed by 10-min H_2_O incubation. Heat-induced antigen retrieval was carried out for 10 min in a pressure cooker in citrate buffer (10 mM Citric Acid, pH 6.0). For IHC, samples were incubated in 1% H_2_O_2_ for 15 min to block endogenous peroxidase activity. Permeabilization was performed using 0.1% normal goat serum in 0.4% Triton-X 100 in PBS for 30 min. Sections were blocked for 1 h at room temperature (RT) with blocking solution (Abcam cat# AB64226; Cambridge, UK) containing M.O.M. blocking reagent (Vector Laboratories cat# MKB2213–1; Newark, CA), incubated with primary antibodies (Sup. Table. 3) at 4°C overnight in antibody diluent (Abcam cat# 64211; Cambridge, UK), then washed 3 times for 30 minutes each in 0.1% triton-X 100 in PBS. For IHC samples, sections were incubated in ImmPRESS HRP goat anti-rabbit or rat IgG polymer detection kit (Vector Laboratories cat# MP-7451/MP7404; Newark, CA) and ImmPACT DAB substrate, Peroxidase HRP (Vector Laboratories cat# SK4105; Newark, CA) according to the manufacturer’s instructions. IHC slides were mounted using micromount mounting medium (StatLab, cat# MMC0126; McKinney, Texas). For IF, sections were incubated with secondary antibodies for 1 h at RT in antibody diluent (Abcam cat# 64211; Cambridge, UK). Sections were then washed in 0.1% Triton-X 100 in PBS 3 times for 30 min each and were mounted using fluoroshield mounting media with DAPI (Abcam cat#104139). Immunofluorescence images were collected using a Zeiss Axiovert 200m fluorescence microscope. IHC images were collected using a Keyence BZ-X800 microscope. Sections staining, image capturing, and image analysis were done manually using ImageJ and were carried out by a researcher who was blinded to sample identities. Subsequent grouping and graphing were done by a different lab personnel unblinded after image analyses and quantification were completed.

### Bronchoalveolar Lavage Fluid (BALF) processing

Bronchoalveolar lavage (BAL) was performed with 1 mL of PBS (ThermoFisher cat#14190–144; Waltham, MA) after mice were euthanized. BALF was collected and centrifuged at 500 × g for 5 min at 4°C. Supernatant was ash frozen in liquid nitrogen and stored at −80°C until analysis. Cells were resuspended in FACS buffer (PBS with 2% FBS and 2 mM EDTA) and were counted manually.

### Flow cytometric analyses

Cells recovered from BALF were stained with antibodies (Extended Data Table. 3). Alternatively, whole lungs were harvested and digested using the method described elsewhere^17^. Briefly, lung digestion mix (1.5 mg/mL collagenase A (Sigma Aldrich cat# COLLA-RO; St. Louis, MO), 0.4 mg/mL deoxyribonuclease I (Worthington cat# LS002139; Lakewood, NJ), 10 mM HEPES pH 7.2, 5% FBS) was injected into the lungs through cannulae and were incubated at 37°C for 30 min. Digested lungs were passed through a 50 mm cell strainer and red blood cells were lysed using hemolytic buffer (150 mM NH_4_Cl, 1 mM NaHCO_3_, 1.1 mM Na_2_EDTA). Single cells were resuspended in FACS buffer and stained with antibodies (Extended Data Table 3) for flow cytometry. Data were collected on the LSR II flow cytometer (BD Biosciences) and analyzed with FlowJo software v10.

### Fixed single cell RNA-seq

Cells exhibiting >80% viability were fixed in a 4% formaldehyde solution and using the Chromium Next GEM Single Cell Fixed RNA Sample Preparation Kit (10X Genomics, Pleasanton, CA). The whole transcriptome probe pairs (10X Genomics) were added to the fixed single cell suspensions to hybridize to their complementary target RNA during an overnight incubation at 42°C. After hybridization, unbound probes were removed by washing. The fixed and probe-hybridized single cell suspensions were loaded onto a Chromium X (10X Genomics) microfluidics instrument to generate partitioned nanoliter-scale droplets in oil emulsion. The target is for each droplet to contain a barcoded gel bead, a single cell, and enzyme Master Mix (10X Genomics) for probe pair ligation and gel bead primer barcode extension. The droplets in oil emulsion were placed in a thermal cycler for 60 min at 25°C, 45 min at 60°C, and 20 min at 80°C. The single cell-barcoded, ligated probe products underwent library preparation using standard 10X Genomics protocols in preparation for Illumina next-generation sequencing. The gene expression library derived from single cell-barcoded, ligated probe product were sequenced as paired-end 150 bp reads on the Illumina NovaSeq 6000 (Illumina, San Diego, CA) at the University of Colorado Genomics Shared Resource (Aurora, CO, USA) at a target depth of 20,000 reads per cell for all samples.

### Data Processing for single cell RNA-seq analysis

Single cell RNA-seq (scRNAseq) fastq files were processed using Cell Ranger software (version 7.1.0)^18^ from 10X genomics to assign reads to genes based on Cell Ranger’s Chromium mouse transcriptome probe set (version 1.0.1). The counts were analyzed using the Seurat R package^19^ and cells with less than 100 genes and genes that were seen in fewer than 10 cells were excluded. The R package scDblFinder^20^ was used to identify and subsequently remove doublets from the data. Based on the distribution of UMIs, gene counts, and percentage of mitochondrial reads, data were filtered to remove cells with fewer than 200 and more than 7,500 UMIs or genes detected and mitochondrial reads greater than 2.5%. The data were then log transformed and scaled, regressing out cell cycle difference score, total UMI and % mitochondrial reads.

Principal Component Analysis (PCA) was performed using the top 2,000 variable genes. PCs (N=30) that captured most of the variation were then included in further data processing steps. Clusters were identified (at a resolution of 1.8) using the K-nearest neighbors’ algorithm. The top 100 enriched genes (with an adjusted P < 0.05 and higher average fold changes) within each cluster and within each sample were used for performing over-representation analysis (ORA)^21^ with gene sets from the MSigDB C8 collection^22^, PanglaoDB^23^, MSigDB^24^, GO biological processes^25^ and Hallmark KEGG pathways^26^ databases, annotated using enriched gene sets and expression of canonical cell type markers. Differentially expressed genes (DEGs) were identified using the Wilcoxon Rank Sum test within each of the cell types identified for the indicated comparisons. Gene set enrichment analysis (GSEA) was performed using the clusterProfiler R package (v 4.6.2) for the indicated comparisons and within cell types of interest. Benjamini-Hochberg method was used to calculate the adjusted P values. Raw and processed scRNAseq data will be deposited to the Gene Expression Omnibus.

### Influenza virus RNA quantification

Whole lung tissue was homogenized, and RNA was isolated via TRIzol/chloroform extraction per the manufacturer’s protocol (ThermoFisher; Waltham, MA and MilliporeSigma; St. Louis, MO, respectively). RNA was reverse transcribed with an iScript cDNA synthesis Kit (Bio-Rad Laboratories, Inc., Hercules, CA) and the viral load was determined by qPCR for the PR8 acid polymerase (PA) gene compared to a standard curve of known PA copy numbers as previously described^27^.

### Quantification and statistical analyses (mouse models)

**Figure 1:**
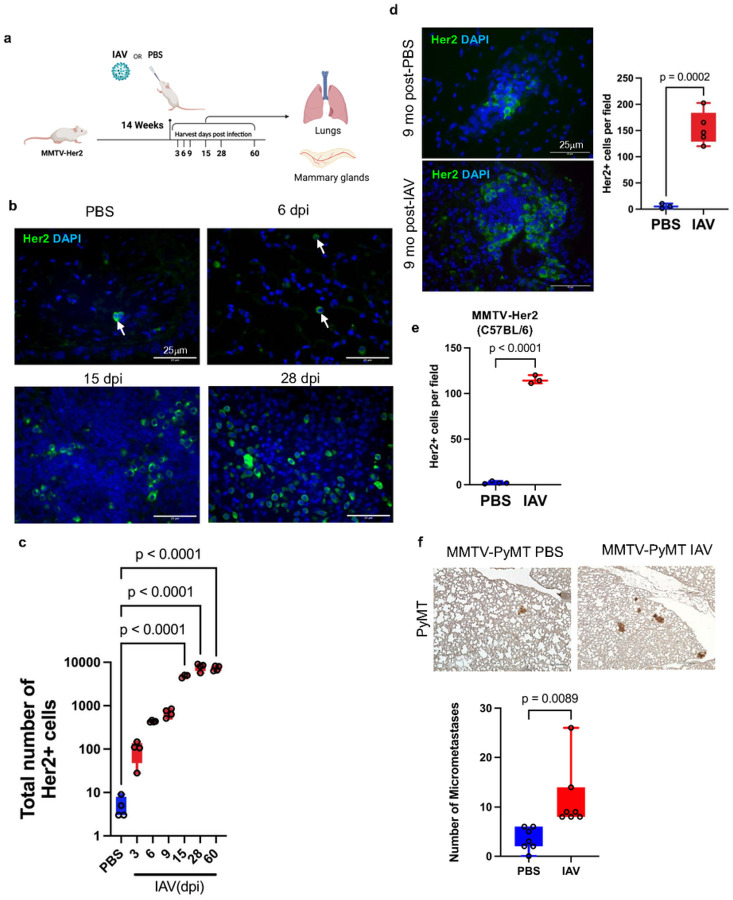
Influenza A virus infection increases DCC in lungs. MMTV-Her2 female mice (N=3–5/group) in FVB background were infected with a sublethal dose of Puerto Rico A/PR/8/34 H1N1 influenza A virus (IAV) by intranasal administration. Lungs and mammary glands were harvested at timepoints indicated post-infection (a) Immunofluorescence and quantification of Her2^+^ cells in lungs 3, 6, 9, 15, 28, 60 dpi, with total numbers from three sections of the whole lung quantified. (b, c) Lung sections were stained with DAPI (blue), and Her2 (green) as a marker for DCC (b). Immunofluorescence and quantification of Her2^+^ cells in lungs 9 months post-influenza infection (d). Quantification of Her2^+^ cells in C57BL6/J MMTV-Her2 mouse lungs at 15dpi with IAV (N=4 PBS, 3 IAV) (e). Immunohistochemistry and quantification of PyMT^+^ micrometastases defined by lesions with an area < 0.03 mm^2^ (N=7/group) (f). Significance determined by one-way ANOVA test.

**Figure 2:**
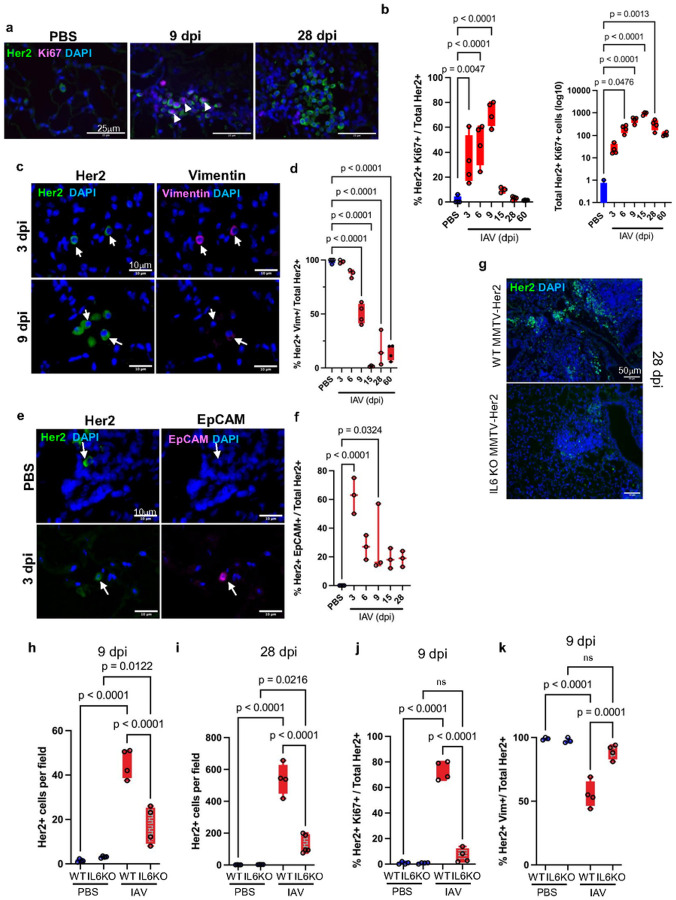
Influenza A virus infection promotes dormant DCC proliferation and induces phenotypic changes through IL-6. Immunofluorescence and quantification of Ki67^+^/Her2^+^ cells in lungs post-influenza A virus (IAV) infection. Lung sections from naïve and IAV-infected mice were stained with antibodies against Her2 (green) and Ki67 (magenta), and DAPI (blue) (a): percentage of Ki67^+^/Her2^+^ cells (b, left) and absolute number of Ki67^+^/Her2^+^ cells across three lung sections (b, right). Immunofluorescence and quantification of vimentin^+^ (Vim^+^) and EpCAM^+^Her2^+^ cells in lungs post-influenza infections where lung sections from naïve and IAV-infected mice were stained with Her2 (green) and vimentin (magenta) (c) or Her2 (green) and EpCAM (magenta) (e), and percentage of vimentin^+^/Her2^+^ or EpCAM^+^/Her2^+^ cells graphed (d, f). Lung sections of MMTV-Her2 or IL-6 KO:MMTV-Her2 mice 28dpi (PBS or IAV) were stained for Her2 (green) and DAPI (blue) (g) and quantified at 9- and 28dpi (h, i). Quantification of the percentage of Ki67^+^Her2^+^ cells in MMTV-Her2 and IL-6 KO:MMTV-Her2 9dpi (j). Quantification of the percentage of vimentin^+^/Her2^+^ cells in MMTV-Her2 and IL-6 KO:MMTV-Her2 mice at 9- and 28dpi (k). 3–5 mice were used in each group. Statistical Significance relative to PBS samples are shown, as determined by one-way ANOVA test.

Statistical analyses were performed using Prism 10.2.1 software (GraphPad). Investigators were not blinded to allocation during virus (IAV or SARS-CoV-2) inoculation or antibody treatment. Quantification and image analysis were done in a blinded manner. N indicates number of mice per group. A minimum of 3 slides per mouse were used for image analysis. Total Her2^+^ cell counts (Fig. 1c, Fig. 2b), Her2^+^ cells, and Her2^+^ Ki67^+^ cells were counted manually using ImageJ. Three lung sections at least 50 μm apart per mouse were counted and summed. For other image quantifications, whole lung images were divided into fields using ImageJ’s grid function, 8–10 fields were selected at random per image and counted. For experiments with two groups, a two tailed student t-test was used; for experiments with more than one group, one-way ANOVA tests were used unless otherwise stated. Data were expressed as mean ± standard deviation (s.d.). P-values ≤ 0.05 were interpreted as evidence against the null hypothesis (i.e. no effect, no difference).

### Human Observational Data

For the human follow-up studies, we selected SARS-CoV-2 infections as the driver virus as SARS-CoV-2 infection due to mandatory reporting during the first phases of the pandemic provides an opportunity to utilize real-world data to investigate the hypothesis that respiratory viral infections promote metastatic disease. We used two complementary datasets: 1) The UK Biobank, a population-based study including 502,356 adult volunteers aged 40 to 69 years at recruitment from 2006 to 2010^28,29^. 2) Breast cancer patients/survivors from the Flatiron Health’s nationwide electronic health record (EHR)-derived database^30,31^.

### Study 1. Population-based analyses in the UK Biobank

We used data from the UK Biobank, a population-based study including 502,356 volunteers aged 40 to 69 years at recruitment from 2006 to 2010^28,29^. Participants provided data on lifestyle, anthropometry, exposures, sociodemographic factors, medical history, and medications at baseline. Previous cancer diagnoses were obtained through (consented) linkage to the national cancer registry and confirmed SARS-CoV-2 test positivity status through linkage to national registers. Mortality data were obtained from the national death registries (NHS Digital, NHS Central Register, National Records of Scotland). We considered all-cause mortality, non-COVID-19 mortality (by excluding deaths with ICD codes U07.1 and U07.2^32^ or any death within one month of the latest recorded test positive result), and cancer mortality (considering cause of death with ICD codes listed in Extended Data Table 1).

As summarised in Figure 4a, we included 502,356 participants, excluding those who withdrew from the study. We excluded 65,374 participants due to missing data on covariates including sex, age, BMI, ethnicity, smoking status, alcohol consumption, education, employment status, and household income (N= 63,557); missing date on SARS-CoV-2 testing when the primary cause of death was COVID-19 (N=129); and missing cancer diagnosis date if the primary cause of death was cancer (N=1,688), leaving 436,982 participants. Of these, 91,518 had been diagnosed with cancer at the latest follow-up (1^st^ June 2022). Overall, 22,597 participants had died before the start of the COVID-19 pandemic (here defined as 1^st^ January 2020) (N=14,974) or were diagnosed with cancer after that date (N=9,543) and were therefore excluded. Participants diagnosed with multiple cancers before baseline were also excluded (N= 6,654).

Of the 60,347 participants with a cancer diagnosis before 1^st^ January 2020, a total of 3,462 had been reported to have tested positive for SARS-CoV-2. To ensure that test-positive and test-negative participants had similar cancer risk profiles, we adopted a non-parametric matching (without replacement) approach^33^ to identify (up to) 10 test-negative participants for each test-positive participant. We first matched for the cancer diagnosis date (prior to 1^st^ January 2020) using generalised full matching^34^. In a second step, we performed an exact matching based on cancer type and sex. We finally matched for age, ethnicity, smoking status, alcohol consumption, education, employment status, and household income using the nearest neighbour method, an algorithm based on propensity score matching. All the matching steps were without replacement. The resulting matched population included 28,855 participants: 3,400 test-positives matched to 25,455 test-negatives. Restricting this matched population to those who tested positive before vaccine rollout (here defined as 1^st^ December 2020) resulted in a total of 7,705 participants including 916 test positives and 6,789 matched test negatives.

Using test positivity as the predictor, we ran a series of unconditional logistic regression models for the three outcomes (all-cause, non-COVID-19, and cancer mortality). Models were adjusted for all matching factors to account for possible residual confounding. As a sensitivity analysis, we considered participants with cancer diagnosed at least five or ten years before the start of the COVID-19 pandemic in the UK by excluding the test-positive participants (and their matched test-negative controls) who were diagnosed with cancer between 1^st^ January 2015 and 31^st^ December 2019 (N=538 test positives and N=4,107 test negatives), and between 1^st^ January 2010 and 31^st^ December 2019, respectively (N=316 test positives and N=2,437 test negatives).

#### Data availability:

This study used the UK Biobank resource under application number 69328 granting access to the corresponding UK Biobank genetic and phenotype data. The UK Biobank received ethical approval from the North West Multi-centre Research Ethics Committee (REC reference: 11/NW/0382) to obtain and disseminate participant data and samples (http://www.ukbiobank.ac.uk/ethics/).

### Study 2. Flatiron - EHR-based Analyses

#### Data source

Flatiron Health’s nationwide electronic health record (EHR)-derived database includes deidentified data from ~ 280 US cancer clinics (~ 800 sites of care). The database is longitudinal, comprising de-identified patient-level structured and unstructured data, curated via technology-enabled abstraction^30,31^. The majority of patients in the database originate from community oncology settings; relative community/academic proportions may vary depending on study cohort. Institutional Review Board approval of the protocol was obtained prior to study conduct and included an informed consent waiver.

Included in our study were women aged at least 18 years old at the time of initial cancer diagnosis, and who had:

1. Early Breast Cancer. The cohort includes a probabilistic sample of patients with a diagnosis of Stage I - III Breast Cancer on or after January 1, 2011, including those who presented with non-metastatic disease but who subsequently developed recurrent or progressive disease, with at least two visits occurring on or after January 1, 2011 or -
2. Metastatic Breast Cancer. The cohort includes a probabilistic sample of patients diagnosed with Stage IV breast cancer on or after January 1, 2011, and those who presented with earlier stage breast cancer but who subsequently developed metastatic disease on or after January 1, 2011, and who had at least two clinic encounters evident in the database occurring on or after January 1, 2011.
3. Adult female patients ages 18+ years at the initial diagnosis

#### Real-world data source:

The index date was defined as the date of the initial diagnosis of breast cancer. The COVID-19 status was defined positive if any COVID diagnosis (ICD codes B97.29, B97.21, J12.81, B34.2, U07.1) was made after the index date, and before the diagnosis of lung metastases or the last follow-up date. The start date of COVID-19 positivity status was the earliest COVID-19 diagnosis date. Baseline characteristics of gender, race, ethnicity, and age at index date were obtained from structured data.

#### Analyses:

Baseline characteristics were summarized using descriptive statistics. Cause-specific analysis was conducted (death was censored). Univariable and multivariable Cox Proportional Hazard Models were used to evaluate the effect of COVID-19 diagnosis on the risk of metastasis to the lungs, in which COVID-19 diagnosis status was treated as a time varying covariate. The multivariable model adjusted for patient characteristics considered relevant, including age, race ethnicity, and gender. The unadjusted and adjusted hazard ratio with the corresponding 2-sided 95% confidence interval were reported. The two-sided likelihood ratio tests were conducted. The significant level was 0.05. Time to metastases to the lungs was defined as time from index date to date of metastases to the lungs. Patients without a date of pulmonary metastases were censored at the last confirmed activity date or death. Last confirmed activity was defined as the latest date of vitals record, medication administration, or reported laboratory tests/results. The statistical analyses were conducted using R version 4.1.0^35^.

## Results

### Influenza A virus infection awakens dormant breast cancer cells in lungs.

The metastatic process of cancer cells involves shedding, intra/extravasation, escaping immunosurveillance, establishment of dormancy at distant sites, and eventually awakening of dormant disseminated cancer cells (DCC)^36,37^. To study the effects of influenza virus infection on awakening of dormant breast DCC in the lung, we used the MMTV-erbB2/neu/HER2 mouse model (hereafter MMTVHer2) where mice overexpress rat Neu (*Erbb2*; paralog of human Her2) in epithelial mammary gland cells, a well-established mouse model of breast cancer metastatic dormancy^14,38^. Her2^+^ early lesion cells in the mammary glands seed the lungs and other organs with DCCs within 12–14 weeks of life where they remain largely as dormant single cells for up to one year before progressing to overt metastatic disease^39^. Thus, this model recapitulates the persistence of dormant DCCs in lungs and bone marrow in individuals who remain in remission for years to decades.

MMTV-Her2 mice (FVB background) were infected with a sublethal dose of influenza A virus (IAV) (Fig. 1a). Infected mice lose weight and recover by day 11–12 days post infection (dpi) (Extended Data Fig. 1a), and wild type (WT) and MMTV-Her2 mice elicit a similar inflammatory response, with increased cellularity of bronchoalveolar lavage (Extended Data Fig. 1b). The kinetics of viral clearance were similar between WT and MMTV-Her2 mice, where IAV RNA copies peaked around 6 dpi, with a 100–1000-fold reduction in viral load from 9–15 dpi (Extended Data Fig. 1c), suggesting that the presence of early DCCs in the lung does not curtail the initial virus-induced inflammatory response.

Lungs of MMTV-Her2 mice (FvB background) were harvested 3, 6, 9, 15, 28, and 60 dpi (Fig. 1a) and examined for the abundance of Her2^+^ cells (Fig. 1b, c) as reported^38^. Consistent with previous work^38,40^, we observed a small number of isolated DCC or small clusters (<10 cells) in the lungs prior to IAV infection. Strikingly, metastatic burden increased 100–1000-fold between 3 dpi to 15 dpi, the number of pulmonary Her2^+^ cells remained elevated even at 28 dpi and 60 dpi (Fig. 1b, c), and the large increase in metastatic burden was still evident at 9 months (Fig. 1d). IAV-mediated expansion of Her2^+^ DCC was similarly observed in the lungs of MMTV-Her2 mice in the C57BL/6J background at 15 dpi (Fig. 1e). Notably, the resultant expanded Her2^+^ cells exhibit a diffuse non-epithelial like architecture in the lungs, unlike the epithelial-like clusters and metastasis (>100 cells/cluster) of DCC observed in the lungs of MMTV-Her2 mice that are >10 months old (Extended Data Fig. 1d and^38,40^).

We performed similar infection experiments in MMTV-PyMT transgenic mice that express polyoma virus middle-T oncoprotein in the mammary gland and also show early dissemination but a shorter-term dormancy in the lungs^40^. MMTV-PyMT mice demonstrated an increased number of small tumor clusters in the lungs following influenza A virus infection (Fig. 1f). All together, these studies show that influenza virus infection can promote DCC expansion in multiple models of breast cancer DCC dormancy.

### IAV-triggered IL-6 induces DCC awakening and phenotypic transitions

When we examined the proliferation of DCC in the lungs, we found a significant increase in the percentage of Her2^+^ cells expressing Ki67 (a marker of all cycle phases except G0) beginning at 3 dpi and peaking at 9 dpi (Fig. 2a, b). Although the fraction of Her2^+^ cells that express Ki67 decreased by 15 dpi, the total *number* of Her2^+^ cells expressing Ki67 remained highly elevated relative to baseline even 60 dpi given the overall increase in DCC burden in the lungs (Fig. 2b). These results indicate that, following IAV infection, DCC in the lungs experience a period of awakening leading to an increase in metastatic burden.

Dormant DCC in the Her2+ and PyMT models are characterized by a ZFP281-driven mesenchymal-like state and express genes such as vimentin, while loss of ZFP281 allows the DCC to adopt a more epithelial-like phenotype characterized by the expression of epithelial markers such as EpCAM and E-cadherin when exiting dormancy^40^. Consistent with previous results, most dormant DCC present in uninfected lungs expressed vimentin and not EpCAM (Fig. 2c–f). The percentage of Her2^+^ cells expressing vimentin was not significantly affected early after infection (3–6 dpi). However, at 9 dpi the percentage of Her2^+^ cells expressing vimentin was decreased to ~50%, with a further decrease to <20% at 28 dpi (Fig. 2d). In contrast, early during IAV infection (3 dpi), a substantial fraction of Her2^+^ cells acquired EpCAM expression, associated with the awakening of DCC (Fig. 2e, f). This increase was largely transient: most Her2^+^ cells were EpCAM negative after 6 dpi, although the percentage of EpCAM^+^Her2^+^ cells remained elevated compared to uninfected lungs (Fig. 2f). Thus, while the loss of the mesenchymal marker is sustained after infection, there is a transient acquisition of a more epithelial phenotype, and the population remains mixed at subsequent timepoints. In all, these data indicate that viral infection is signaling to dormant DCC to attenuate the mesenchymal-like state and adopt a hybrid state that allows for dormant DCC awakening.

Inflammatory cytokines such as IL-6 and IL-1 are known to promote cancer malignancy and metastases^41–43^. In addition, IL-6 produced during acute inflammation resulting from biopsy or chemotherapy contributes to the development of lung metastatic outgrowth of disseminated mammary tumor cells ^9,10^. IL-6 (but much less IL-1β) is abundantly produced during IAV infection, in part due to the replication of the virus in lung epithelial cells^44^. Similarly, we also detected high levels of IL-6 in bronchoalveolar lavage fluid (BALF) from wild-type and MMTV-Her2 mice following IAV infection, with very low levels of IL-1β (Extended Data Fig. 2a, b).

To determine whether IL-6 production triggered by IAV infection contributes to the awakening of dormant DCC, we used MMTV-Her2 mice crossed with IL-6 KO mice in the FVB background^15^. IL-6 KO:MMTV-Her2 mice were infected with IAV and harvested at 9 and 28 dpi (at this dose of IAV, all mice recovered without excessive weight loss). Prior to infection, there was no difference in the number of dormant Her2^+^ cells between IL-6 KO:MMTV-Her2 and MMTV-Her2 lungs (Fig. 2h, i), and these mice developed primary tumors requiring sacrifice with similar timing at older ages (Extended Data Fig. 3a). Thus, IL-6 is not required for primary tumor growth or for early cancer cell dissemination to the lungs. Strikingly, the number of Her2^+^ cells in lungs of IAV-infected IL-6 KO:MMTV-Her2 mice was drastically decreased compared with infected MMTV-Her2 mice at both 9 and 28 dpi (Fig. 2h, i), with substantial reductions in Ki67^+^Her2^+^ cells (Fig. 2j). Similarly, in the MMTV-PyMT mouse model of breast cancer metastasis, IAV-induced proliferation of PyMT^+^ small lesions and formation of micro-metastases in the lungs was dampened by IL-6 deficiency (Extended Data Fig. 3b, c). Staining for vimentin demonstrated that most Her2^+^ cells in lungs of IL-6 KO:MMTV-Her2 mice retain vimentin expression, supporting a dampened conversion from dormancy to awakening (Fig. 2k). These results indicate that IL-6 triggered by IAV infection plays a key role in mediating dormant DCC reawakening, proliferation, and phenotypic changes.

### CD4 T cells are required for maintenance of expanded DCC post-IAV infection.

**Figure 3:**
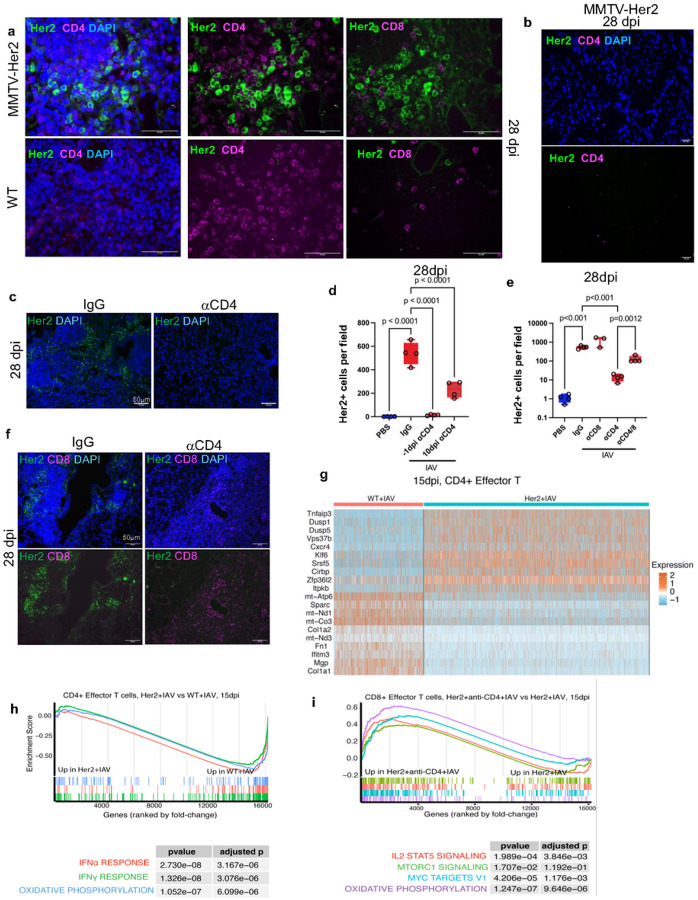
CD4^+^ cells are required to maintain IAV-mediated Her2^+^ DCC expansion. Adjacent lung sections of IAV-infected mice 28dpi were stained for Her2 (green) and CD4 (red, middle) or Her2 (green) and CD8 (red, right) (a). As in (a) for IAV-infected MMTV-Her2 mice 28 dpi, but for a region devoid of CD4 cells (b). Lung sections of MMTV-Her2 or CD4-depleted-MMTV-Her2 mice 28 dpi were stained for Her2 (green) and DAPI (blue) (c). Number of Her2^+^ cells were quantified from IAV-infected mice with CD4 depleted −1 and 10dpi (d). Quantification of Her2^+^ cells from MMTV-Her2 mice with CD4, CD8, CD4/8 depletion (on −1dpi) at 28dpi (e). Lung sections of MMTV-Her2 and CD4-depleted MMTV-Her2 mice at 28dpi were stained for Her2 (green), CD8 (magenta), and DAPI (blue) (f). 3–5 mice were used in each group. p determined by one-way ANOVA test. Heatmap of top 20 differentially expressed genes from scRNAseq comparing CD4 effector T cells from MMTV-Her2+IAV versus WT+IAV mice at 15 dpi (g). GSEA analyses comparing effector CD4 T cells in MMTV-Her2+IAV versus WT+IAV mice at 15 dpi (h), and effector CD8^+^ T cells in MMTV-Her2+IAV versus CD4-depleted MMTV-Her2+IAV mice at 15 dpi (i).

While IL-6 was essential for the awakening and the initial marked expansion of dormant DCC, minimal levels of IL-6 were detected in BALF of MMTV-Her2 mice 15 dpi (Extended Data Fig. 2a), suggesting the presence of other factors that promote survival post-expansion of DCC at later times post-infection. While recruitment of neutrophils to the lung occurs by 3 days post-infection with IAV, CD4 T cells, CD8 T cells, and B cells accumulate in the lung from around day 9 dpi in both WT and MMTV-Her2 mice (Extended Data Fig. 4a-d). Infection with IAV has also been shown to induce the formation in the lungs of inducible bronchus-associated lymphoid tissues (iBALT), lymphoid organizations that include primarily CD4 cells together with B cells. iBALT can be detected in the lungs long after the infection (up to 100 dpi)^45^. Accordingly, we also detected these CD4 cell and B cell-enriched lymphoid organizations in the lung sections of WT and MMTV-Her2 mice 28 dpi (Fig. 3a; Extended Data Fig. 4f, g). As expected, B cells in these iBALT are also positive for the germinal center B cell marker GL7. In contrast to CD4 cells, very few CD8 cells were present in these lymphoid structures in either WT or MMTV-Her2 mice (Fig. 3a). Interestingly, co-staining of CD4 with Her2 revealed the selective presence of DCC in proximity to high-density clusters of CD4 cells. Regions lacking CD4 cells also lack Her2+ cells (Fig. 3b).

These results suggested that CD4 cells could be contributing to the maintenance of awakened DCC for longer periods post-IAV infection. We therefore examined the effect of depleting CD4 cells by administration of an anti-CD4 antibody to MMTV-Her2 mice starting the day prior to IAV infection (day −1) (Extended Data Fig. 5). Analysis of Her2^+^ cells in the lung sections 28 dpi revealed a drastic decrease of the number of DCC in anti-CD4-treated mice relative to control IgG-treated mice (Fig. 3c–e), demonstrating the contribution of CD4 cells to the maintenance of DCC post-awakening with influenza virus infection. In contrast, CD4 cell depletion (day −1) did not affect the numbers of Her2^+^ cells at 9 dpi (Extended Data Fig. 6a, b), consistent with the delayed accumulation of CD4 cells late during the infection (Extended Data Fig. 4b). Notably, the number of Her2^+^ cells in the lung was also significantly decreased when the depletion of CD4 cells was initiated 10 dpi (Fig. 3d), supporting the contribution of CD4 cells later during the infection. Together these data show that IL-6 (but not CD4 cells) contributes to the initial awakening and expansion of dormant DCC, but that later during the infection following the recruitment of T cells, CD4 cells are required for the maintenance of the awakened DCC.

Previous studies have shown how neutrophil extracellular traps produced during inflammation can awaken dormant cancer cells in the lungs^46^. However, in contrast to CD4 cell depletion, the depletion of neutrophils with an anti-Ly6G antibody at the time of IAV infection did not alter the numbers of Her2^+^ cells in the lung (Extended Data Fig. 6c-e). Similarly, depletion of CD8 cells at the time of the infection had no effect on the presence of DCC (Fig. 3e), consistent with the paucity of CD8 cells in the lungs 28 dpi (Fig. 3a). Thus, maintenance of the awakened lung DCC following IAV infection is selectively dependent on the presence of CD4 cells.

Interestingly, while only a low number of dispersed CD8 cells were present in the lungs 28 dpi in MMTV-Her2 mice, we found an increased accumulation of CD8 cells in the lungs of infected mice when CD4 cells were depleted (Fig. 3f). These results suggested that CD4 cells may repress the recruitment of CD8 cells to the lung, thus potentially compromising the immune surveillance against the awakened DCC. We therefore tested the effect of depleting both CD4 and CD8 cells on the maintenance of lung DCC following IAV infection. While CD4 cell depletion results in a marked reduction of Her2^+^ cells in the lungs of infected mice, the dual depletion of CD8 cells and CD4 cells partially restored the numbers of Her2^+^ cells in the lung (Fig. 3e). Thus, the effect of CD4 cells in the maintenance of awakened DCC in the lung post-IAV infection is in part mediated by repressing CD8 cell mediated immune responses.

We then examined whether the presence of awakened DCC in the lungs following IAV infection could reprogram the recruited T cells to a more suppressive or suppressed state by performing single cell RNA-seq (scRNAseq) of lungs of WT and MMTV-Her2 mice 9 and 15 dpi with IAV, at points where the accumulation of T cells in the lungs is high (Extended Data Fig. 7a-d). As expected, multiple immune cell types such as macrophages, natural killer (NK) cells, B cells, effector CD4 cells, CD4 IL7-receptor+ cells, effector CD8 cells, and proliferating effector CD8 cells were present in the lung at both 9- and 15 dpi (Extended Data Fig. 7b). We had two replicates for most conditions, which exhibited very similar gene expression patterns (Extended Data Fig. 7e, f). IAV infection induced type I and II interferon (IFN) responses across these cell types, as expected (Extended Data Fig. 8). We examined the phenotype of effector CD4 cells in WT and MMTV-Her2 mice. Interestingly, there was substantially increased expression of a selective subset of genes in effector CD4 cells from MMTV-Her2 mice relative to effector CD4 cells in WT mice. In particular, Tnfaip3, Zfp36l2, Dusp5, Dusp1, Klf6, Pdcd4, and Ctla4 were highly upregulated in effector CD4 cells from MMTV-Her2 mice relative to WT mice (Fig. 3g and Extended Data Resource 1). Tnfaip3 (which encodes the E3 ubiquitin ligase A20) suppresses anti-tumor activity of CD8 T cells^47^, whilst Zfp36l2 (which regulates RNA stability through AU-rich elements) restrains CD8 cell activation and expansion^48,49^. Dusp5 (which encodes a dual phosphatase) suppresses T cell proliferation and promotes their survival^50^. Klf6 and Dusp1 are markers of central and resident memory T cells ^51,52^. Ctla4 induces Pdcd4 in cytotoxic T cells, and *Pdcd4* deficiency enhances their anti-tumor effector functions^53^. In addition, comparing memory-like CD4^+^IL7R^+^ cells^54^ from Her2^+^ versus WT mice at day 15 after IAV infection revealed similar changes in gene expression, with increased Klf6, Tnfaip3, Dusp1, Dusp10, and Pdcd4 in the infected Her2^+^ mice (Extended Data Fig. 8a). These data suggest that CD4 cells from infected MMTV-Her2 mice have more of a memory type phenotype than effector cells with increased survival and less effector function.

In addition, CD4 cell expression of genes important for T-cell activation, including Gadd45b and Slfn2^55–57^, was reduced in CD4^+^ effector cells in infected Her2+ mice relative to WT mice (Fig. 3g and Extended Data Resource 1). Interestingly, the expression of a number of mitochondrial genes (e.g. mt-Atp6, mt-Nd1, mt-Co3, mt-Nd3) was greatly reduced in CD4 cells from MMTV-Her2 mice (Fig. 3g, Extended Data Fig. 8a and Extended Data Resource 1), indicating a reduced mitochondrial content in these cells. The reduced mitochondrial content could result from increased autophagy mediated by the upregulation of Tnfaip3, since Tnfaip3 deficiency has been shown to increase mitochondrial content in CD4 cells by promoting autophagy^58^, or from reduced CD4 cell growth.

Pathway analysis further supports a compromised effector function of CD4 cells from MMTV-Her2 mice. Over-Representation Analysis (ORA) of scRNAseq comparing MMTV-Her2 and WT mice after IAV infection showed reduced interferon responses in MMTV-Her2 mice across multiple innate immune cell types and B cells, consistent with dampened increases in types I and II IFN (Extended Data Fig. 8c, d, 9a). Gene expression in CD4 cells was also strikingly different, with a significant suppression of multiple activation-associated pathways including IFN pathways, Cytokine-Cytokine Receptor Interaction, and Oxidative Phosphorylation in effector CD4 cells from infected MMTV-Her2 mice relative to infected WT mice by 15 dpi (Fig. 3h). Similarly, comparisons between CD8^+^ cells from WT and MMTV-Her2 mice after IAV infection showed a reduction in these pathways in the presence of DCC (Extended Data Fig. 8b, 10). Taken together, these results suggest that the presence of DCC impairs CD4 and CD8 cell activation in response to IAV infection, favoring tumor cells persistence.

Critically, GSEA comparing CD8^+^ T cells from MMTV-Her2 mice with IAV infection and CD4 depletion revealed significant activation of pathways involved in CD8^+^ cell activation 15 dpi such as IL2/STAT5 and MTORC1 signaling pathways, suggesting that CD8^+^ cells are more proliferative and activated when CD4 cells are depleted (Fig. 3i). Thus, the effect of CD4 cell depletion on eliminating DCC appears to be mediated by enhanced CD8 cell responses against DCC.

### SARS-CoV-2 virus infection, DCC awakening and cancer-specific death in humans.

**Figure 4:**
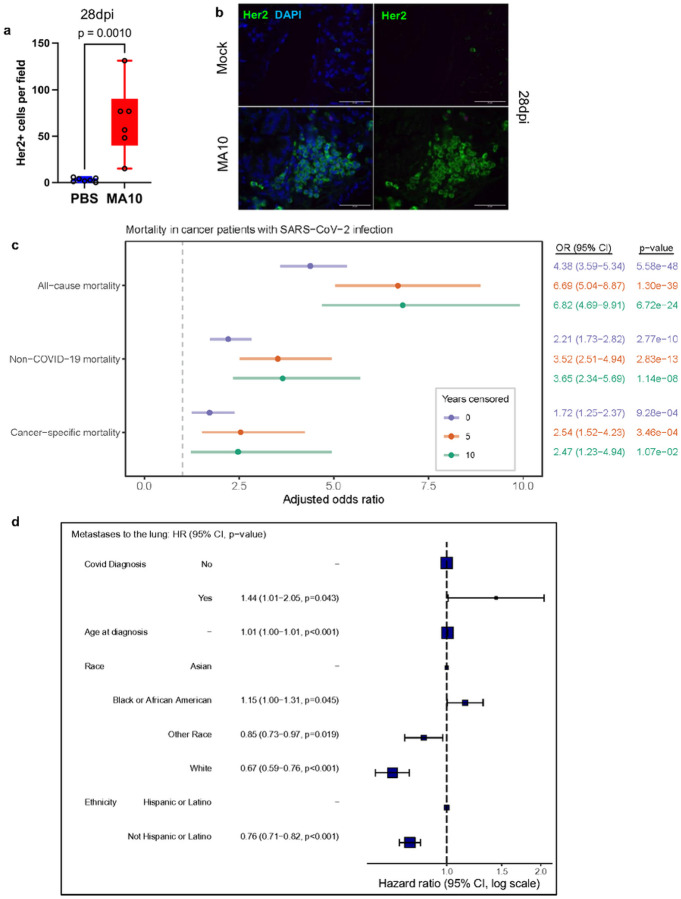
SARS-CoV-2 infection increases cancer progression and metastasis to lungs. Quantification of Her2^+^ cells (a) across three lung sections in C57BL6/J MMTV-Her2 mouse lungs at 28dpi with MA10 SARS-CoV-2 (N=6) or vehicle (PBS; N=7). Immunofluorescent stain of Her2 at 28dpi (b). Analyses of UK Biobank for the association of cancer patients tested positive for SARS-CoV-2 or not and the risk of cancer-related mortality (c). Analyses of the Flatiron Health database with the hazard ratio of risks of progression to metastatic disease in the lungs between breast cancer patients tested COVID-positive or not (d).

To determine whether SARS-CoV-2 infection of lungs can promote the reawakening of dormant DCC, we performed studies (analogous to those in Fig. 1 with IAV) using a mouse adapted SARS-CoV-2 that recognizes mouse ACE2, termed MA10, obtained by genetic modification of the spike gene^59^ followed by serial passages in mice^16^. This SARS-CoV-2 strain induces a COVID-19 like disease in mice including acute lung injury characterized by impaired pulmonary function, diffuse alveolar damage and infiltration of immune cells^16^. Infection of MMTV-Her2 (C57BL6/J) mice with MA10-SARS-CoV-2 resulted in a striking increase in Her2^+^ cells by 28 dpi (Fig. 4a–b).

We utilized the COVID-19 pandemic, as a unique opportunity study the effect of pulmonary virus infections on cancer progression. In contrast to influenza, data on virus infections and resulting disease has been systematically collected in the first years of the pandemic. First, we analyzed data from the UK Biobank to determine whether a SARS-CoV-2 positive test result, among a population of cancer survivors, was associated with an increased risk of cancer-related mortality (Extended Data Fig. 11). We limited the analysis to subjects with a positive test prior to December 2020, to mitigate potential confounding factors from the deployment of vaccines and the increasing use of at-home COVID tests, as well as to provide an adequate follow-up period to assess mortality rates. Among our full study population including 7,705 participants with a cancer diagnosis before 1^st^ January 2020, we observed a total of 606 deaths (418 in the test negatives and 188 in the test positives), yielding an OR of 4.38 (95% CI 3.59–5.34) (Fig. 4c). When excluding the 95 deaths directly attributed to COVID-19, our analysis showed an increased mortality in COVID-19 test positive cases (OR of 2.38:95% CI 1.85–3.06). Using the 49 cancer-related deaths as outcome, we estimated a close to two-fold increase (OR 1.79 (95% CI 1.29–2.48)) in cancer mortality in participants with a prior cancer diagnosis who tested SARS-CoV-2 positive compared to those who tested negative. To focus on patients in inferred remission, we excluded participants whose latest cancer was diagnosed within five or ten years of the start of the COVID-19 pandemic in the UK. Analyses of cancer cases at least 5 years before a potential infection (i.e. diagnosed prior to January 1^st^ 2015; N=4,645, N deaths all-cause=276, N non-COVID deaths = 210, N cancer deaths=96) revealed an increased all-cause (OR= 6.68; 95% CI 5.03 −8.86), non-COVID-19 (OR= 3.59; 95% CI 2.55–5.06) and cancer mortality (OR=2.42; 95% CI 1.44–4.06) between test positives and test negatives (Fig. 4c). Analyses based on the participants diagnosed with cancer prior to January 1^st^ 2010 (N=2,753, N deaths all-cause=158, N deaths non-COVID-19, and N cancer-related deaths=52) showed an increase in (i) all-cause mortality (OR=6.79; 95% CI 4.67–9.87), (ii) non-COVID-19 mortality (OR=3.72; 95% CI 2.37–5.85), and (iii) cancer mortality (OR=2.29; 95% CI 1.13–4.63) in test positives compared to test negatives. Stratification of results based on primary tumor type and on metastatic disease was not possible due to insufficient numbers of observations. We can only infer that patients 5 or 10 years out from initial diagnosis of cancer are likely in remission (and thus any residual metastatic cancer cells are likely dormant). Recognizing these limitations, these data reveal a striking increased risk of death from cancer for cancer survivors who experience SARS-CoV-2 infection.

We utilized the Flatiron Health database with 36,845 female breast cancer patients with complete information to determine whether women with a primary diagnosis of breast cancer experienced an increased risk of progression to metastatic disease in the lungs following COVID-19 (Extended Data Table 2A; Extended Data Fig. 12). Critically, female breast cancer patients who experienced COVID-19 after their initial diagnosis exhibited a hazard ratio (HR) of 1.44 (p=0.043) for subsequent diagnosis of metastatic breast cancer in the lungs (Figure 4d and Extended Data Table 2b-e). These data indicate that COVID-19 increases the risk of progression to lung metastasis for female breast cancer patients.

## Discussion

**Figure 5:**
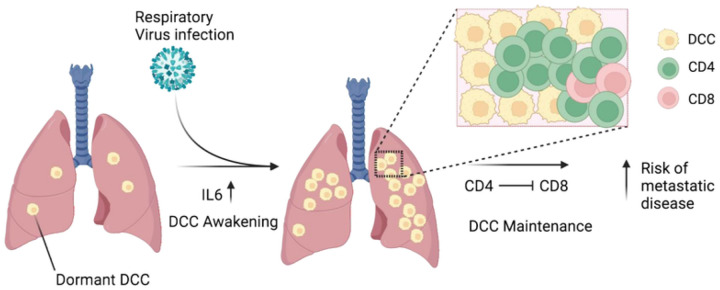
Model. Pulmonary virus-dependent increases in IL-6 contribute to the awakening and expansion of dormant mesenchymal-like breast cancer cells that switch to a partial epithelial-like phenotype in lungs in the early phase of viral infection. CD4 cells maintain the expanded breast cancer cells in late phase of viral infection through suppressing CD8 cells. Virus-dependent awakening and expansion of DCC in the lungs increases the risks of metastatic progression.

Our results indicate that respiratory virus infections promote the awakening and expansion of dormant cancer cells that had seeded the lungs before viral infection. This proceeds through two phases. First, an IL-6 dependent switch of DCC from a mesenchymal phenotype to a hybrid state that promotes expansion. Second, this expansion is followed by a significant cessation of proliferation and the establishment of CD4 cell niches that inhibit DCC elimination in part through suppression of CD8 cells (Fig. 5). We further reveal how the presence of Her2^+^ tumor cells results in a suppressive phenotype for CD4+ cells, and that depletion of CD4^+^ cells leads to elimination of influenza virus infection expanded DCC, dependent on CD8^+^ cells. We further show that a mouse-adapted SARS-CoV-2 similarly leads to DCC expansion in the lung.

Since early in the pandemic, the research community has pondered how COVID-19 might influence cancer pathogenesis^60–63^. The results of the UK-biobank analyses show that individuals with a prior diagnosis of cancer are at increased risk of dying of cancer after having tested positive for SARS-CoV-2. The cause of the increased cancer mortality is not known, but a re-activation of dormant cancer cells may play a role, which is bolstered by the significant increased risk of cancer deaths for individuals who were 5 or 10 years out from their cancer diagnoses. Greater specificity was obtained for analyses of the Flatiron Health database, demonstrating a substantial increase in the risk of progression to metastatic lung disease for women previously diagnosed with breast cancer who experienced COVID-19. Together with our mouse models, these results reveal the substantial risk for cancer survivors who experience COVID-19.

FDA-approved strategies for managing severe COVID-19 include the inhibition of IL-6^64^ or downstream (through JAK1/2^65^) signaling, and modern cancer therapies frequently leverage checkpoint inhibitors to reinvigorate adaptive immunity in patients with cancer, raising the prospect of interventions that could reduce the risk of respiratory virus infection-induced metastatic cancer progression. Of course, such an intervention would need to be safe, or even beneficial to patients with a virus infection (as IL-6/JAK inhibitors can be), to justify application for the likely millions of cancer survivors who experience respiratory virus infections.

In all, our studies should have significant implications for understanding how infections could impact the risks of cancer recurrence, and inform public and medical policy on how to limit the increased risks for lung metastases resulting from COVID-19 or other respiratory virus infections.
